# Suppression of Wnt/β-catenin Signaling in PDAC via METTL16-mediated N6-methyladenosine Modification of DVL2

**DOI:** 10.7150/jca.85860

**Published:** 2023-09-11

**Authors:** Lanting Yu, Jiawei Lu, Ni Xie, Lutong Fang, Sumin Chen, Ying Wu, Xingpeng Wang, Baiwen Li

**Affiliations:** 1 Department of Gastroenterology, Shanghai General Hospital, Shanghai Jiao Tong University School of Medicine, Shanghai 201620, China.; 2 Shanghai Key Laboratory of Pancreatic Diseases, Shanghai General Hospital, Shanghai Jiao Tong University School of Medicine, Shanghai 201620, China.; 3 The First Affiliated Hospital of Anhui Medical University, Anhui 230022, China.; 4 Department of Gastroenterology, Jiading Branch of Shanghai General Hospital, Shanghai Jiao Tong University School of Medicine, Shanghai 201803, China.

**Keywords:** METTL16, Pancreatic ductal adenocarcinoma, Wnt/β-catenin signaling, m^6^A, DVL2

## Abstract

Pancreatic cancer is a formidable cause of cancer-related deaths worldwide and has witnessed a more than twofold increase in incidence over the last 25 years. The most frequently occurring form of pancreatic cancer is pancreatic ductal adenocarcinoma (PDAC), accounting for the majority of pancreatic cancer cases. N6-methyladenosine (m6A), the most abundant transcript modification, has been implicated in the pathogenesis of numerous human cancers, including pancreatic cancer. Despite this, the functional role of methyltransferase-like 16 (METTL16), a critical m6A methyltransferase, in PDAC remains elusive. In this study, we demonstrate that METTL16 expression is significantly diminished in PDAC, rendering it a promising prognostic indicator. Strikingly, both in vitro and in vivo assays revealed accelerated metastasis and invasion of PDAC cells upon METTL16 knockdown, while overexpression of METTL16 exerted an opposite effect. Mechanistically, METTL16 regulates DVL2 expression by suppressing its translation via m^6^A modification, thereby regulating Wnt/β-catenin signaling., Our results unveil the downregulation of METTL16 as a concomitant increase in DVL2 levels via m6A modification promoting the progression of PDAC. Thus, we propose METTL16 as a novel therapeutic candidate for targeted PDAC treatment.

## Introduction

Approximately 60,000 new cases of pancreatic ductal adenocarcinoma (PDAC) are diagnosed each year, and nearly half of them have advanced stages at diagnosis, with a five-year survival rate of 10% for the first time in 2020^1^. The causes behind its poor prognosis are a lack of early effective screening, rapid cancer progression, early metastasis, and poor treatment efficacy caused by high chemoresistance^2^. Therefore, to improve the prognosis of PDAC, early diagnosis and therapeutic targets are essential. Epithelial-mesenchymal transition (EMT) is a crucial step in metastasis, which deprives epithelial cells of a polarized organization while gaining migratory and invasive capabilities^3^. During EMT, epithelial markers (e.g., E-cadherin and ZO-1) are downregulated or lost, while mesenchymal markers (e.g., N-cadherin) are upregulated^4^, ^5^. It is now widely recognized that EMT is relevant to the invasion, dissemination, and metastasis of epithelial cancers, pancreatic cancer included. The mechanisms behind this process are complex, including epigenetic changes^6^, ^7^.

Dysregulation of Wnt signaling pathway is a key factor of EMT in PDAC^8^. According to the Wnt/β-catenin pathway, β-catenin is phosphorylation and degradation by a destruction complex composed of AXIN, APC and GSK3β. However, when Wnt ligands bind to their receptors, they trigger the recruitment of AXIN and the destruction complex to the plasma membrane by DVL proteins, leading to the inhibition of β-catenin phosphorylation and degradation. Consequently, β-catenin accumulates in the cytoplasm and enters the nucleus, where it activates the transcription of oncogenic and metastatic genes such as c-myc, and MMP7^9^. Epigenetic modifications of Wnt pathway components affect the tumor initiation and progression in PDAC, indicating its therapeutic value as an epigenetic target for PDAC^10^, ^11^.

More than 150 RNA modifications have been identified as post-transcriptional regulatory regulators that regulate alternative splicing, RNA export, RNA stability, as well as, translation. Moreover, their dysregulation has been linked to cancer's occurrence and progression^12^. As a reversible epigenetic modification, N^6^-methyladenosine (m^6^A) modifications are prevalent and are found in approximately one-third of mammalian mRNAs, with an average of three to five m^6^A modifications per mRNA^13^. To date, the multicomponent m^6^A methyltransferase complex (MTC) mainly composed of METTL3 and METTL14 is regarded as the main writer and has been extensively explored. However, in some cell types, the deletion of METTL3 and METTL14 resulted in a reduction of only approximately 60% in m^6^A modification, and a significant number of m^6^A markers did not map within the binding sites of METTL3 and METTL14. Hence, we hypothesize that other methylases are involved and play an indispensable role in the m^6^A modification of RNA. In mammals, both METTL3 and METTL16 are m^6^A methyltransferases that regulate mRNA methylation. Unlike METTL3, which forms an m^6^A methyltransferase complex, METTL16 appears to function as a monomer^14^. The function of METTL16 in cells is just beginning to be understood. Currently, the research uncovered that METTL16 interacts with MALAT1 lncRNA, U6 snRNA, and MAT2A pre-mRNA, whereas only the latter two were confirmed to be methylated via METTL16. Additionally, in hepatocellular carcinoma, METTL16 was identified to promote translation in an N6-methyladenosine (m^6^A)-independent manner^15^-^18^. In PDAC, patients exhibiting elevated levels of METTL16 demonstrated enhanced responsiveness to poly-(ADP-ribose)-polymerase inhibitor therapy by modulating MRE11 nuclease activity in an m^6^A-independent fashion^19^. However, whether METTL16 is associated with the formation and progression of PDAC by regulating the m^6^A level of RNA has not been explored previously.

In this study, we demonstrate that METTL16 is downregulated in PDAC and that METTL16 depletion exerts a stimulative effect on metastasis and invasion of PDAC by activating Wnt/β-catenin signaling. Moreover, we confirmed that METTL16 mediates the m^6^A methylation of DVL2 to regulate the Wnt/β-catenin signaling pathway. Overall, we describe a novel METTL16/DVL2-β-catenin signaling regulation mechanism and provide potential prognostic and diagnostic indicators in PDAC.

## Methods

### Cell lines and patient tissue samples

Human pancreatic cancer cell lines: PANC-1, SW-1990, BxPC-3, and AsPC-1 were purchased from the Type Culture Collection of the Chinese Academy of Sciences (Shanghai, China), and normal pancreatic ductal epithelial cells (HPDE6-c7) were obtained from the Beijing North Carolina Chuanglian Biotechnology Research Institute (Beijing, China). Among them, PANC-1 and MIA PaCa-2 cells were cultured in Dulbecco's modified Eagle's medium (HyClone) supplemented with 10% fetal bovine serum (Gibco). Moreover, other cell lines were grown in RPMI-1640 medium (Gibco) with 10% fetal bovine serum (Gibco). All cells were maintained at 37°C with 5% CO_2_ in a humidified incubator. PDAC tissues were obtained as mentioned previously 20.

### Tissue microarray

Tissue microarrays (TMAs) were obtained from 79 PDAC patients. Moreover, written informed consent was obtained from all patients. The use of clinical samples was approved by the Ethics Committee of Shanghai General Hospital (approval number: 2020SQ050).

### Reverse transcription-quantitative PCR and western blot

Reverse transcription-quantitative PCR (RT-qPCR) and western blot methods were described previously 20. The oligos and primers used in this study were listed in table S1. Moreover, the primary antibodies used in this study were as follows: GAPDH (cat. no. 2118, 1:1,000; Cell Signaling Technology), histone H3 (cat. no. 4499t, 1/1,000; Cell Signaling Technology), METTL16 (cat. no. A15894, 1/1,000; ABclonal), E-cadherin (cat. no. 20874-1-AP, 1/10,000; Proteintech), N-cadherin (cat. no. 66219-1-Ig, 1/5,000; Proteintech), ZO-1 (cat. no. 21773-1-AP, 1/5,000; Proteintech), DVL2 (cat. no. 12037-1-AP, 1/5,000; Proteintech), β-catenin (cat. no. A19657, 1/1,000; ABclonal), Phospho-β-catenin-S45 (cat. no. AP0580, 1/1,000; ABclonal), METTL3 (cat. no. A8370, 1/1,000; ABclonal), and METTL14 (cat. no. 26158-1-AP, 1/1,000; Proteintech).

### Transwell cell migration and invasion assays

Cell mobility and invasiveness were evaluated by using 8-μm-pore Transwell inserts (Corning, NY, USA) without (migration) or with (invasion) Matrigel (BD Biosciences, NY, USA). Briefly, cells were resuspended in a serum-free medium and placed into the upper chamber of the Transwell unit. The lower chambers were added with 10% fetal bovine serum. After incubation, the upper chamber cells were removed, and the lower chamber cells were fixed in 4% paraformaldehyde, stained with 0.1% crystal violet solution, and counted in five random fields under a microscope.

### Wound-healing assay

Cell monolayers were seeded into six-well plates. When the cells reached 100% confluence, the cell layers were scratched with a pipette tip. Next, cells were cultured in a serum-free medium for 24 h (AsPC-1 cells were incubated in 1% fetal bovine serum for 72 h) and imaged under a microscope.

### Lentivirus transfection

Lentiviruses were purchased from Shanghai Genechem. Cells were seeded into 6-well plates. And they were transduced with the appropriate lentiviruses when cells reached 50% confluence in the presence of HitransG A (Shanghai Genechem). We selected transduced cells using 2 ug/ml puromycin (Beyotime Institute of Biotechnology) and assessed transfection efficiency by RT-qPCR and western blotting.

### H&E staining

Fresh tissues were fixed in 4% paraformaldehyde at room temperature for 24 hours and embedded with paraffin. The embedded tissue was sliced into 4 μm thick slices and stained with hematoxylin and eosin (H&E). All images were captured using an optical microscope.

### Dual-luciferase reporter assay

Cells were seeded in 96-well plates and co-transfected with pRL-TK vector, TOP-Flash or FOP-Flash luciferase reporters, along with either sh-METTL16 or METTL16 plasmid post-adhesion. Following a two-day incubation period, the Dual-luciferase reporter assay was conducted utilizing the Dual Luciferase Reporter Gene Assay Kit provided by YEASEN (Shanghai, China), as per the instructions. Varioskan Flash (Thermo Fisher Scientific, USA) was used to detect luciferase activity, after which the ratio of firefly luciferase luminescence to Renilla luciferase luminescence was calculated and analyzed.

### RNA stability assays and protein stability assays

Incubate cells with actinomycin D (MCE, NY, USA) at 5 ug/mL or cycloheximide (CHX) (MCE, NY, USA) at 100 ug/mL and incubation for different times respectively. For the RNA stability assay, total RNA was extracted and detected by RT-qPCR. For the protein stability assay, proteins were measured by western blot.

### Polysome profiling

Cells were treated with 100 µg/mL of CHX for 5 min at 37℃ and 5% CO_2_. Then, cells were washed with ice-cold 1× PBS containing 100 µg/mL of CHX and lysed in lysis buffer (5 mM of Tris-HCl pH 7.5, 2.5 mM of MgCl_2_, 1.5 mM of KCl, 1× protease inhibitor cocktail, 5 μL of 10 mg/mL CHX, 1 μL of 1 M of DTT, 100 units of RNAse inhibitor, 25 μL of 10% Triton X-100, and 25 μL of 10% sodium deoxycholate). Then, lysates were centrifuged at 16,000 g at 4℃ for 7 min. The supernatant was loaded onto the top of a 10~50% sucrose gradient tube and centrifuged at 36,000 rpm for 2 h at 4℃ using an SW 41 Ti rotor. Then RNA in polysome fraction was extracted and subjected to RT-qPCR.

### RNA immunoprecipitation

The interaction between METTL16 and DVL2 mRNA was detected using an RNA immunoprecipitation kit (Geneseed, Guangzhou, China). First, 1 × 10^7^ cells were lysed in lysis buffer and divided into three samples: anti-METTL16, anti-IgG, and input. Second, samples were incubated with anti-METTL16 (cat. no. ab185990; Abcam) or anti-IgG (cat. no. 2729; Cell Signaling Technology) and antibody-conjugated protein A magnetic beads at 4℃ overnight. After purification, RNA was validated using qRT-PCR.

### m^6^A quantification

Total RNA was extracted using TRIzol. 100-300ng of RNA was used for each sample analysis. The m^6^A level in total RNA was evaluated using the EpiQuik^TM^ m^6^A RNA Methylation Quantification Kit (Colorimetric) (Epigentek, NY, USA) according to the manufacturer's instructions.

### m^6^A methylated RNA immunoprecipitation qPCR

Total RNA was isolated as mentioned earlier and fragmented according to the manufacturer's instructions (riboMeRIP m^6^A Transcriptome Profiling Kit). Next, the m^6^A antibody-conjugated magnetic beads A/G were incubated with fragmented RNA at 4 ℃ for 2 h. After washing, the precipitated RNA was validated by qRT-PCR.

### Immunofluorescent staining

Cells were seeded into the confocal plates and were fixed with 4% paraformaldehyde for 30 min. After permeabilization with 0.5% Triton X-100 for 15 minutes, the cells were blocked for 1 h with 5% BSA. Subsequently, they were incubated with a primary anti-MELLT16 antibody (cat. no. A15894, 1/100; Abclonal) at 4℃ overnight, followed by a secondary antibody at room temperature for 1 h away from light. At last, DAPI was added for 10 min to stain the nucleus of cells. Images were observed with a fluorescence microscope (Leica Microsystems GmbH).

### Immunohistochemistry

The cells were probed overnight with an anti-METTL16 antibody (cat. no. A15894, 1:100; ABclonal), followed by 1 h of room temperature incubation with the secondary antibody (cat. no. GB23303, 1:200; Servicebio). Immunohistochemistry (IHC) staining scores based on staining intensity and staining area was evaluated by three independent observers. IHC staining intensity was scored as follows: blank = 0; yellow = 1; dark yellow = 2; and brown = 3. The frequency of positive cells was defined as follows: 0-5% = 0; 6-25% = 1; 26-50% = 2; 51-75% = 3; and 76-100% = 4. The final score was determined by multiplying the staining intensity scores with the positive area scores.

### Immunofluorescent/RNA fluorescence in situ hybridization

Immunofluorescent was performed as mentioned earlier. To illuminate the location of DVL2 mRNA, h-DVL2-FISH-probe was designed and synthesized by Guangzhou RiboBio. A fluorescence in situ hybridization kit (RiboBio, Guangzhou, China) was used according to the manufacturer's protocol. Briefly, cells were fixed with 4% paraformaldehyde for 10 min and washed with PBS. Then permeabilize cells for 5 min at 4℃. After pre-hybridize the samples for 30 min at 37℃, we incubated the samples with the RNA probe overnight at 37℃. The nuclear staining was performed using DAPI after posthybridization washes. Images were captured through a confocal microscope (Leica, Germany).

### Animal models

The hepatic metastasis model was used to detect the function of METTL16 *in vivo*. BALB/c nude mice aged 4 weeks were divided into four groups randomly (n = 3). Subsequently, a longitudinal abdominal median incision was created after the mice were anesthetized. A total of 2.0 x 10^6^ cells were injected into the spleen. Approximately 2.5 months later, liver tissues were harvested, imaged, and subjected to H&E staining.

### Statistical methods

Data were statistically analyzed via GraphPad Prism 7.0. Student's t-test was used to evaluate the statistical significance between the two groups and one-way ANOVA was used to assess the significance among multiple groups. A linear relationship between variables was assumed when calculating Pearson's correlation coefficient. The survival difference of METTL16 was evaluated by the Kaplan-Meier method, log-rank test, and the survival curve. *P* < 0.05 was considered statistically significant.

## Results

### Aberrant expression of METTL16 in PDAC

To investigate the role of METTL16 in PDAC, the expression of METTL16 was first analyzed using the NCBI Gene Expression Omnibus (GEO) datasets. From the GEO databases GSE15471 and GSE16515, it was found that the METTL16 mRNA levels are downregulated in pancreatic cancer (GSE15471, *p* = 0.0202 and GSE16515, *p* = 0.0007) (Figure S1A). To further evaluate the METTL16 expression at the protein level, the HPA database was used to compare the immunohistochemical staining of METTL16 in normal pancreas and PDAC. The results again indicated that METTL16 is downregulated in pancreatic cancer tissues (Figure S1B). Next, through the Kaplan-Meier plotter database^21^, it was found that patients with higher METTL16 expression exhibited better overall survival significantly (Figure S1C).

**Figure 1:**
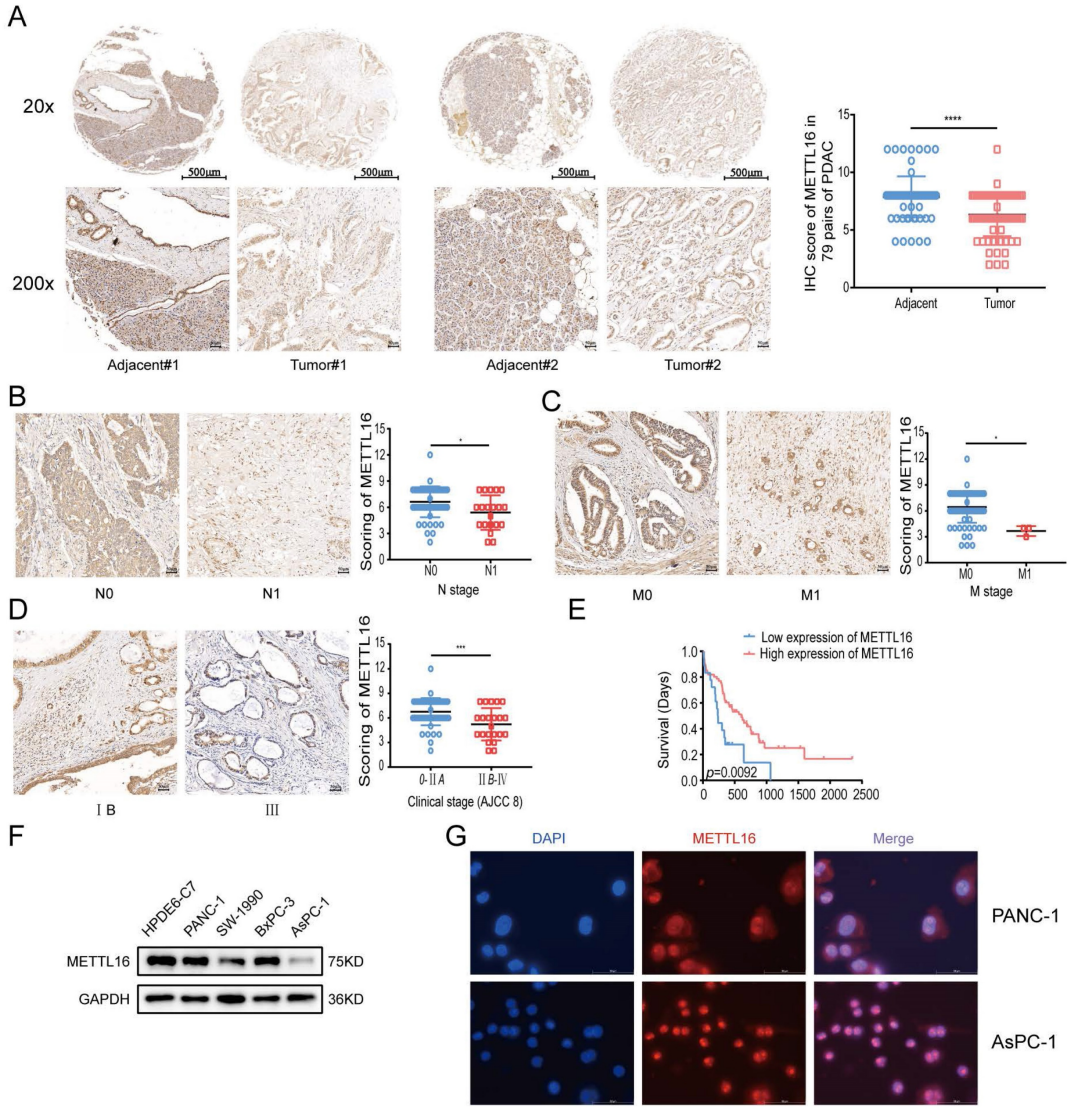
Aberrant expression of METTL16 in PDAC. A Representative IHC images of METTL16 in the TMA (top image scale bar, 500 µm; top image magnification, ×20; bottom image scale bar, 50 µm; bottom image magnification, ×200). B Representative images of IHC of METTL16 in TMA samples of N0 vs N1 stage (scale bar, 50 µm; magnification, ×200). C Representative images of IHC of METTL16 in TMA samples of M0 vs M1 stage (scale bar, 50 µm; magnification, ×200). D Representative images of IHC of METTL16 in TMA samples of AJCC 0-IIA vs IIB-IV stage (scale bar, 50 µm; magnification, ×200). E IHC staining of the TMA determined the overall survival of patients with low and high METTL16 expression levels. F Western blotting was used to determine METTL16 expression levels in pancreatic cancer cells and normal pancreatic ductal epithelial cell (HPDE6-c7). G Representative fluorescence images of the localization of METTL16 protein in PDAC cells (scale bar, 50 µm; magnification, ×630). *P<0.05, **P<0.01, ***P<0.001 and ****P<0.0001. All the data are presented as mean±SD (*Student's t-test*).

Finally, for the verification, a TMA comprising 79 pairs of PDAC samples and PDAC cell lines was used in the subsequent experiments. IHC analysis on TMA demonstrated that METTL16 expression was remarkably lower in PDAC tissues than that in normal adjacent tissues (Figure 1A). We found that METTL16 expression did not correlate with the T stage and pathological grade (Figure S1D and E), whereas tumors with higher N stage or M stage tended to express a lower level of METTL16 (Figure 1B and C) (N stage, *p* = 0.0119; M stage, *p* = 0.0108). Moreover, it was demonstrated that PDAC tissues from the American Joint Committee on Cancer (AJCC) stage IIB-IV have a lower METTL16 level than those from the AJCC 0-IIA (Figure 1D) (*p* = 0.0007). Furthermore, downregulated METTL16 significantly reduced OS in PDAC patients (Figure 1E), which is in line with the results of the Kaplan-Meier plotter database (Figure S1C).

The protein levels of METTL16 in cell lines measured by western blot revealed that METTL16 expression was decreased in PDAC cell lines when compared with that of HPDE6-c7 cell lines (Figure 1F). Among these PDAC cell lines, PANC-1 expressed the highest METTL16 expression, whereas, AsPC-1 expressed the lowest level of METTL16, thus they were selected for the following experiments.

Subsequently, immunofluorescence assays manifested that METTL16 is localized in both the nucleus and cytosol of PANC-1 and AsPC-1 cell lines, but mainly in the nucleus (Figure 1G). In sum, these results indicated that METTL16 is frequently downregulated in PDAC; in addition, its low expression predicted a worse prognosis.

### METTL16 downregulation promotes PDAC metastasis and invasion

**Figure 2:**
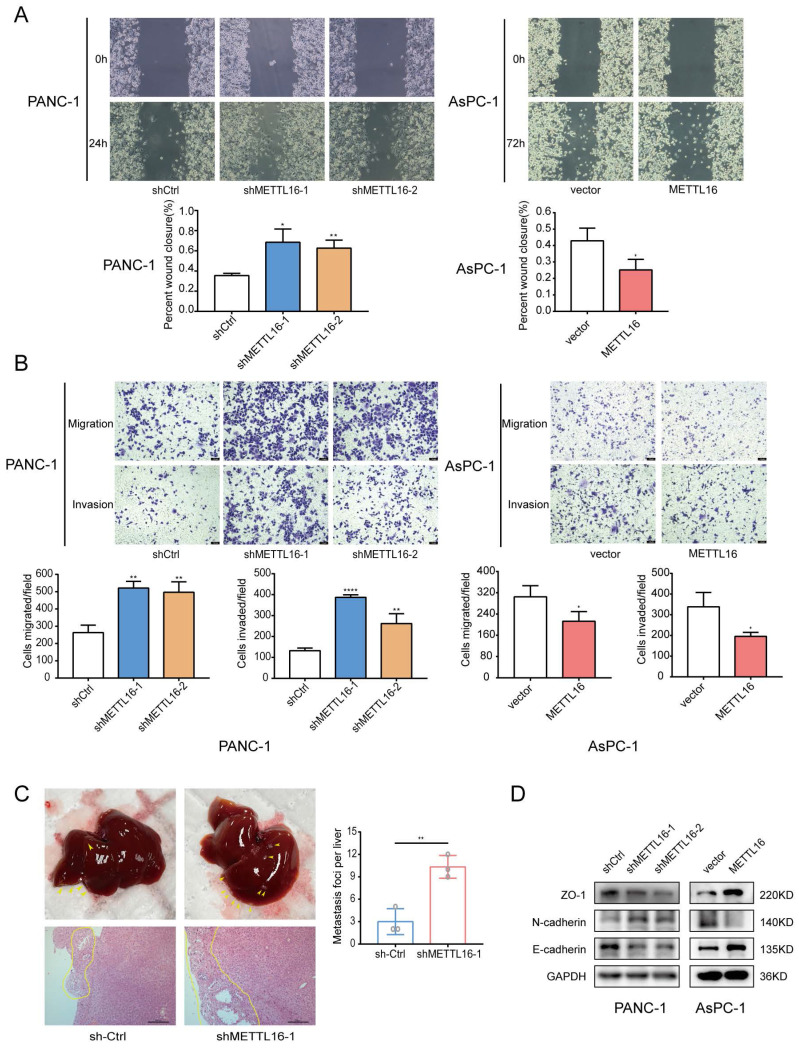
METTL16 impairs PDAC metastasis and invasion. A and B Cell migration and invasion abilities were detected in PDAC cells transduced with shMETTL16 or METTL16 lentiviruses by wound-healing assays and transwell assays. C Representative liver images and H&E staining of the liver tissues of the respective groups. D Western blotting was conducted to analyze the changes in EMT markers in PDAC cells. All experiments were performed in triplicate. *P<0.05, **P<0.01, ***P<0.001, and ****P <0.0001. All the data are presented as mean ± SD (*One-way ANOVA* and* Student's t-test*).

To understand the biological function of METTL16 in PDAC, two shRNAs (sh-METTL16-1 and sh-METTL16-2) and a lentiviral overexpression vector targeting METTL16 were constructed. After transduction, METTL16 expression were detected via RT-qPCR and western blot (Figure S1F). The wound-healing assay and Transwell assays revealed that METTL16 depletion enhanced both the migration and invasion of PANC-1 cells and AsPC-1 cells, whereas overexpression of METTL16 had an opposite effect on PDAC cells (Figure 2A, 2B, Figure S2A and S2B).

To determine the function of METTL16 *in vivo*, PANC-1 cells were chosen to construct stable cell lines (shMETTL16, METTL16, and their corresponding NC). Because shMETTL16-1 was more efficient than shMETTL16-2, shMETTL16-1 was used for the *in vivo* assay. Consistent with the *in vitro* functional experiments results, more liver metastases were found in mice treated with shMETTL16-1 cells (Figure 2C), whereas METTL16 overexpression attenuated liver metastasis (Figure S2C).

Subsequently, the expression of several crucial regulators related to cell migration and invasion was examined. It was demonstrated that METTL16 depletion decreases the expression of epithelial markers: E-cadherin and ZO-1, but increases the expression of N-cadherin (mesenchymal markers). Conversely, overexpression of METTL16 exerts the opposite effect on the expression of E-cadherin, N-cadherin, as well as, ZO-1 (Figure 2D). These results indicated that METTL16 has a vital role in PDAC migration and invasion.

### METTL16 regulates the progression of PDAC cells via the Wnt/β-catenin pathway

To explore the mechanism of METTL16 in PDAC, we obtained a list of METTL16 co-expressed genes in TCGA PAAD from the online database LinkedOmics^22^, and we performed the KEGG pathway analysis on these genes by The Database for Annotation, Visualization, and Integrated Discovery (DAVID)^23^, ^24^. Then it was found that METTL16 may regulate the Wnt signaling pathway Figure S3A).

**Figure 3:**
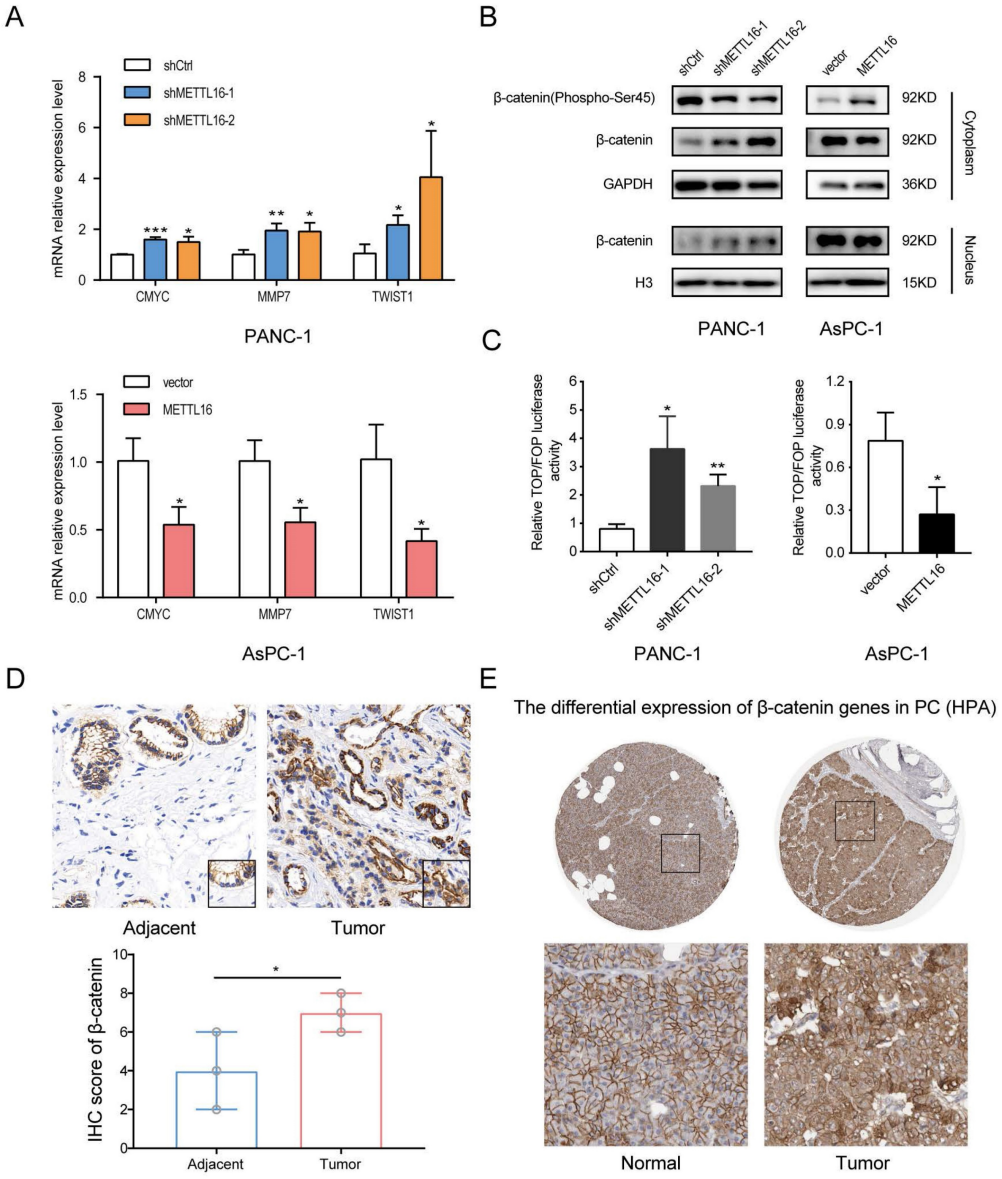
METTL16 inhibits the Wnt/β-catenin signaling pathway in PDAC cells. A RT-qPCR analysis of the expression of the established downstream targets for the Wnt/β-catenin pathway in PDAC cells transduced with siMETTL16, METTL16 overexpression lentiviruses or the corresponding controls. B The effects of METTL16 on the expression of β-catenin (Phospho-Ser45), cytosolic β-catenin and nuclear β-catenin in the indicated cells, as determined by western blotting. C The indicated cells were transfected with TOP or FOP reporter and Renilla pRL-TK plasmids and subjected to dual-luciferase assays after transfection. The detected reporter activity was normalized to the Renilla activity. D Representative images of IHC of β-catenin in PDAC samples. E The protein expression of β-catenin in pancreatic cancer tissue through analysis of HPA databases. All experiments were performed in triplicate. *P<0.05, **P<0.01 and ***P<0.001. All the data are presented as mean ± SD (*One-way ANOVA* and* Student's t-test*).

Considering the importance of Wnt/β-catenin signaling in the progression of PDAC and EMT^8^, ^25^, we were motivated to investigate whether METTL16 regulates this pathway in PDAC. First, we detected the expression of several well-established downstream targets of Wnt/β-catenin signaling in stable knockdown or overexpressed PDAC cell lines. As shown in Fig. 3A, silencing METTL16 promotes the mRNA expression levels of CMYC, MMP7, and TWIST1, whereas overexpression of METTL16 decreases the mRNA expression of these genes. Second, we found that knockdown of METTL16 reduced the expression of Phospho-β-catenin-S45 in the cytoplasm, and promoted the expression of β-catenin of the cytoplasm and nucleus in PANC-1 cells. However, METTL16 overexpression increased the cytosolic level of Phospho-β-Catenin-S45 and decreased the expression of β-catenin of both nuclear and cytoplasm in the AsPC-1 cell line (Figure 3B). To further investigate the impact of METTL16 on Wnt/β-catenin pathway activity, TOP-Flash and FOP-Flash luciferase reporters were employed. As illustrated in Figure 3C, METTL16-knockdown resulted in elevated TOP/FOP luciferase activities in PDAC cells, while transfection with METTL16 overexpressing plasmid led to diminished TOP/FOP luciferase activities. In addition, we detected the protein level of β-catenin in PDAC tissues and corresponding adjacent tissues, and we observed that the expression of β-catenin was elevated in tumor tissues compared to the paracancer tissues, and β-catenin exhibited a differential subcellular localization: it was mainly localized at the cell membrane in the paracancer tissues, whereas it was more distributed in the nucleus and cytoplasm in tumor tissues, which is consistent with the results of HPA database (Figure 3D and E). These observations suggest that METTL16 exerts an inhibitory effect on Wnt/β-catenin pathway activation in PDAC cell lines.

### DVL2 is identified as a downstream target of METTL16

METTL16 inhibits the activation of the Wnt/β-catenin pathway in PDAC, but the mechanism behind it remains ambiguous. Here, we focus on the canonical Wnt/β-catenin pathway components. According to the comprehensive analysis of the prediction results of the RW2Target (a prediction database for targets of RNA modifications)^26^ and the characteristics of the sequence that METTL16 tends to bind, we screened out several key molecules of the Wnt/β-catenin pathway and conducted RNA immunoprecipitation assay to verify their binding. As Figure S4A showed, METTL16 interacts with the mRNA of the dishevelled segment polarity protein 2 (DVL2), so we focused on DVL2 for further investigation.

**Figure 4:**
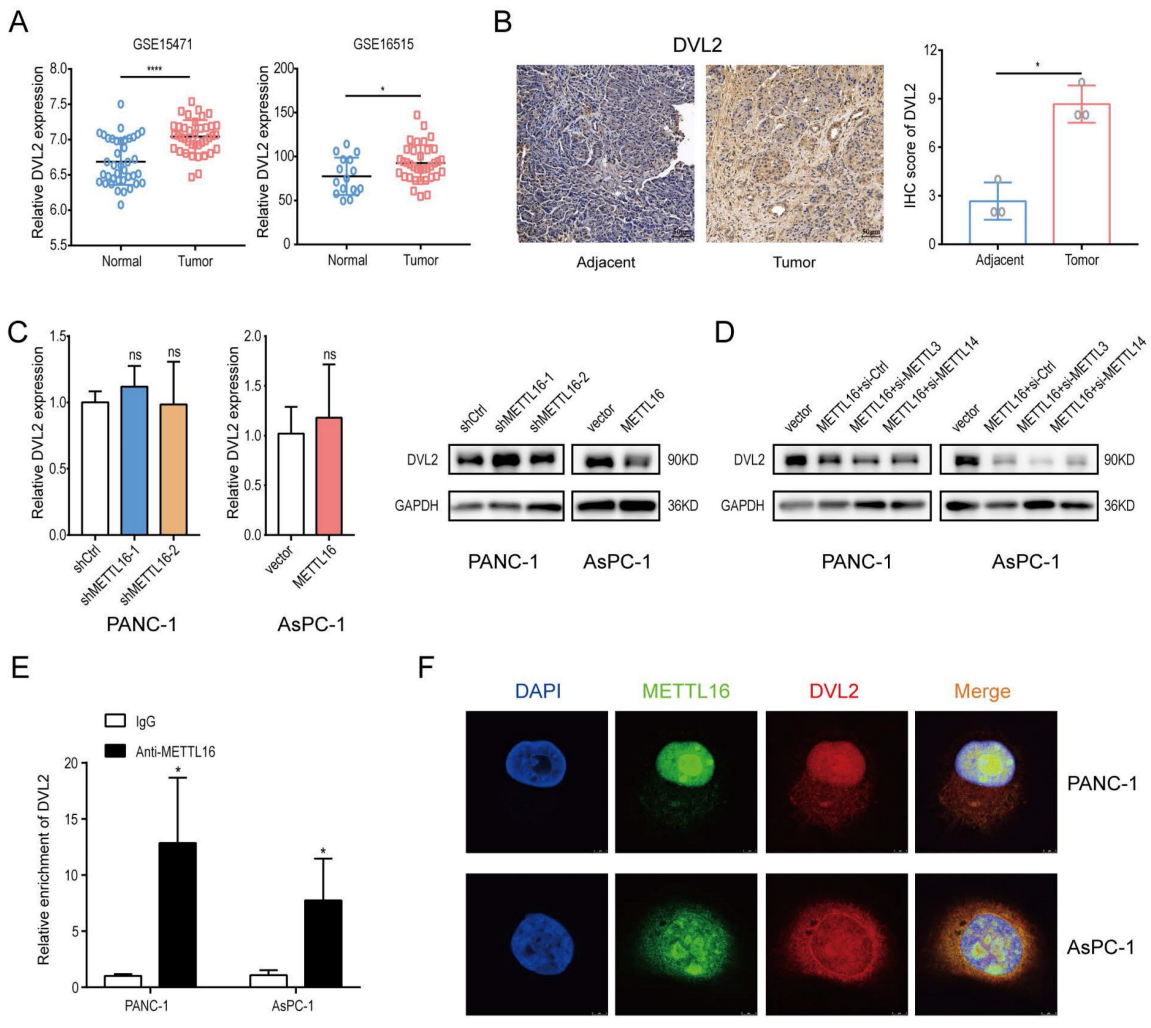
Identification of METTL16 downstream target. A DVL2 Mrna expression levels in Gene Expression Omnibus datasets, GSE15471 (N, n=39; T, n=39) and GSE16515 (N, n=16; T, n=36). B Representative images of IHC of DVL2 in PDAC samples. C RT-Qpcr and western blotting were used to determine DVL2 expression levels following transduction with shMETTL16 or METTL16 overexpression lentiviruses. D Effects of small interfering RNA siMETTL3 and siMETTL14 on DVL2 protein expression in PDAC cells with METTL16 overexpression. E RIP-Qpcr analysis of binding of METTL16 protein to DVL2 Mrna. F IF/FISH was used to identify the location of METTL16 protein and DVL2 Mrna (scale bar: 5 µm; magnification: ×630). *P<0.05, **P<0.01, ***P<0.001, and ****P <0.0001. All the data are presented as mean ± SD (*One-way ANOVA* and *Student's t-test*).

First, the analysis of GEO databases (GSE15471, GSE16515) and IHC staining results of PDAC patients' tissue indicated that the level of DVL2 was elevated in pancreatic cancer (GSE15471, *p* < 0.0001, GSE16515,* p* = 0.0205) (Figure 4A and B).

Second, we evaluated the mRNA level of DVL2 in stable METTL16 knockdown and overexpressed cell lines and found that the mRNA level of DVL2 remained unchanged whether METTL16 was knocked down or overexpressed. Intriguingly, DVL2 protein expression levels were increased markedly after silencing METTL16. In contrast, overexpression of METTL16 leads to the downregulation of DVL2 protein expression (Figure 4C). Because METTL16 has been confirmed to mediate MAT2A to regulate cellular SAM homeostasis, thus exerting its secondary effect on METTL3/METTL14 complex targets^17^, ^27^, we performed a rescue assay to explore whether the aforementioned regulatory relationship exists among METTL16, METTL3/METTL14 complex and DVL2 in PDAC cell lines. Small-interfering RNAs (si-METTL3 and si-METTL14) were transfected into PDAC cells. After transfection, METTL3 and METTL14 expression levels were detected via western blot (Figure S4B). Moreover, we found that silencing METTL3 and METTL14 cannot rescue METTL16 overexpression-induced inhibitory effect on the expression of DVL2 (Figure 4D). To further explore the relationship between METTL16 and DVL2, we performed an RNA immunoprecipitation assay. The results demonstrated that DVL2 specifically combines with endogenous METTL16 (Figure 4E). Meanwhile, immunofluorescence/RNA fluorescence in situ hybridization staining further confirmed the colocalization of METTL16 and DVL2 mRNA (Figure 4F). In sum, these findings suggested the potential regulation of DVL2 expression through METTL16.

### DVL2 mRNA translation is mediated by METTL16

**Figure 5:**
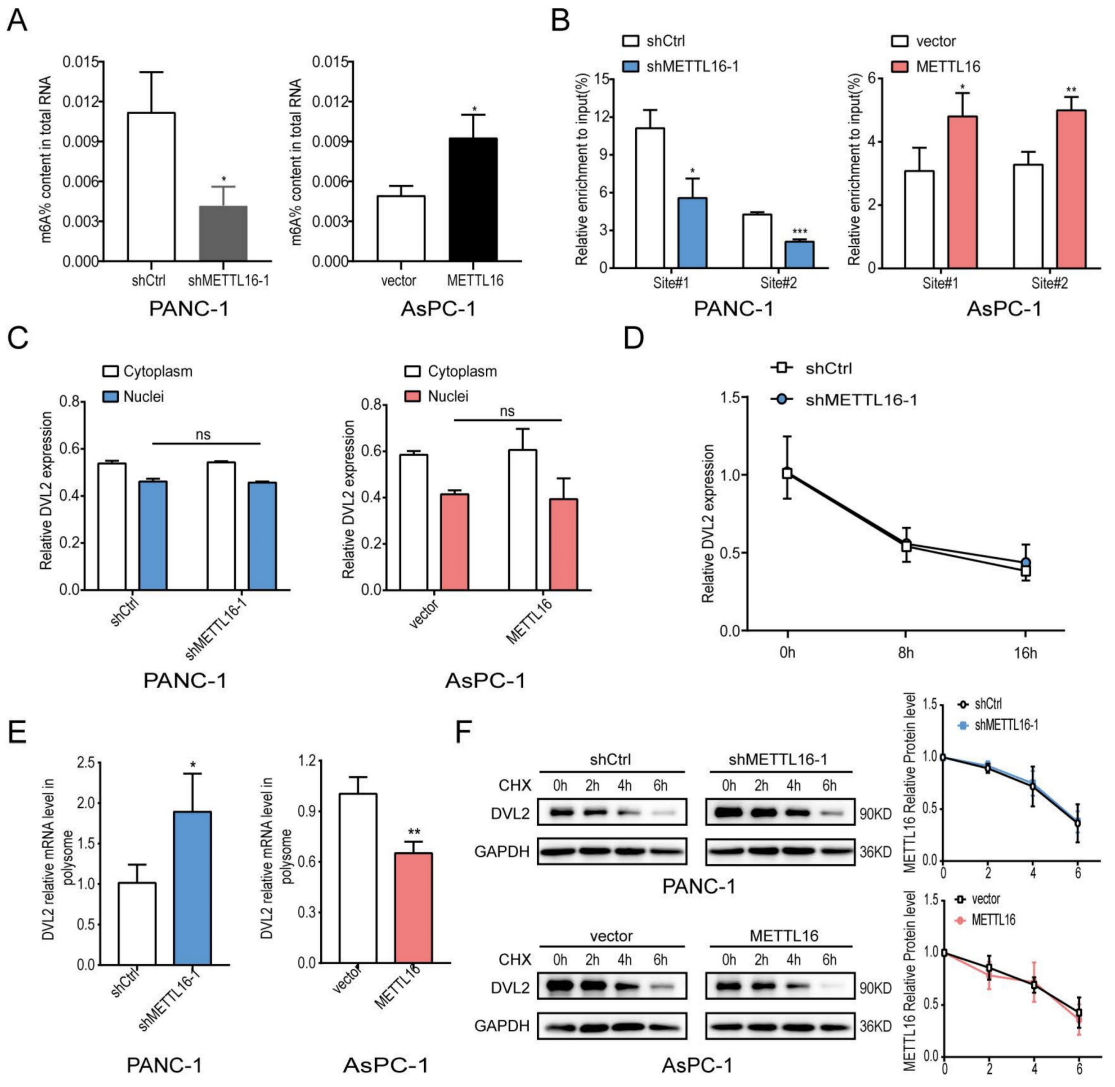
METTL16 inhibits DVL2 translation. A Colorimetric quantification of m^6^A in PDAC cells after METTL16 knockdown or overexpression. B m^6^A enrichment of DVL2 mRNA in PDAC cells was validated by MeRIP-qPCR. C Expression of cytoplasmic or nuclear DVL2 mRNA levels in PDAC cells transduced with METTL16 knockdown or overexpression lentiviruses were detected by RT-qPCR. D DVL2 mRNA stability in indicated PDAC cells was detected by RT-qPCR. E DVL2 mRNA expression of polysomal fractionated RNA in indicated PDAC cells. F DVL2 protein stability in indicated PDAC cells was measured via western blotting. All experiments were performed in triplicate. Data are presented as the mean ± SD (*Student's t-test*). *P<0.05, **P<0.01, and ***P<0.001

The above results have confirmed that METTL16 regulates the expression of DVL2. DVL2 level was uncharacteristically elevated in PDAC, and it was confirmed to promote PDAC progression^28^, ^29^. Since METTL16 is a methyltransferase, we further investigated whether METTL16 regulates DVL2 mRNA through m^6^A methylation. Because shMETTL16-1 was more efficient than shMETTL16-2, shMETTL16-1 was used for the subsequent experiments. First, we examined the m^6^A modification function of METTL16 and found that when we knocked down METTL16, the m^6^A level in PDAC cell lines was down-regulated, and conversely, when we overexpressed METTL16, the m^6^A level in PDAC cells was elevated (Figure 5A). Therefore, we further explore the molecular mechanism between METTL16 and DVL2. A recent publication indicated that “UGAAGA” is the top motif of specific METTL16-bound targets^18^, and we found two such sequences in DVL2 mRNA, one distributed at the CDS regions (site#1), and the other distributed at the 3′UTR regions (site#2). Hence, we designed primers for these two sites to be used in methylated RNA immunoprecipitation (meRIP) experiments. m^6^A MeRIP-qRT-PCR confirmed that the DVL2 m^6^A is decreased upon METTL16 knockdown, whereas forced expression of METTL16 increased the DVL2 m^6^A level (Figure 5B). Our results manifested a reversible m^6^A modification of DVL2 mRNA after the alteration of METTL16 in PDAC. Next, we investigated the effect of METTL16 on DVL2. The fractionation assay indicated that METTL16 did not change the subcellular localization of DVL2 (Figure 5C). In addition, the mRNA stability remained unchanged after the METTL16 was downregulated (Figure 5D). Then, we explored whether METTL16-mediated m^6^A modification affects the translation of DVL2. Our data indicated that polysome-bound (translationally active) DVL2 mRNA levels were significantly elevated in shMETTL16 cells compared with control cells. Conversely, overexpression of METTL16 in AsPC-1 cells reduced the proportion of DVL2 transcripts in polysomal fractions (Figure 5E). Subsequently, the protein stability of PDAC cell lines was assessed by CHX, and we found that DVL2 protein stability was not affected by METTL16 (Figure 5F). In addition, we performed GO analysis on METTL16 co-expressed genes in TCGA PDAC via DAVID database, and found that METTL16 was mainly involved in translation, which was consistent with the above results (Figure S3B). Taken together, these results indicated that METTL16 catalyzes the m^6^A modification on DVL2 mRNA and inhibits its translation.

### DVL2 is critical for the regulation of Wnt/β-catenin signaling by METTL16

**Figure 6:**
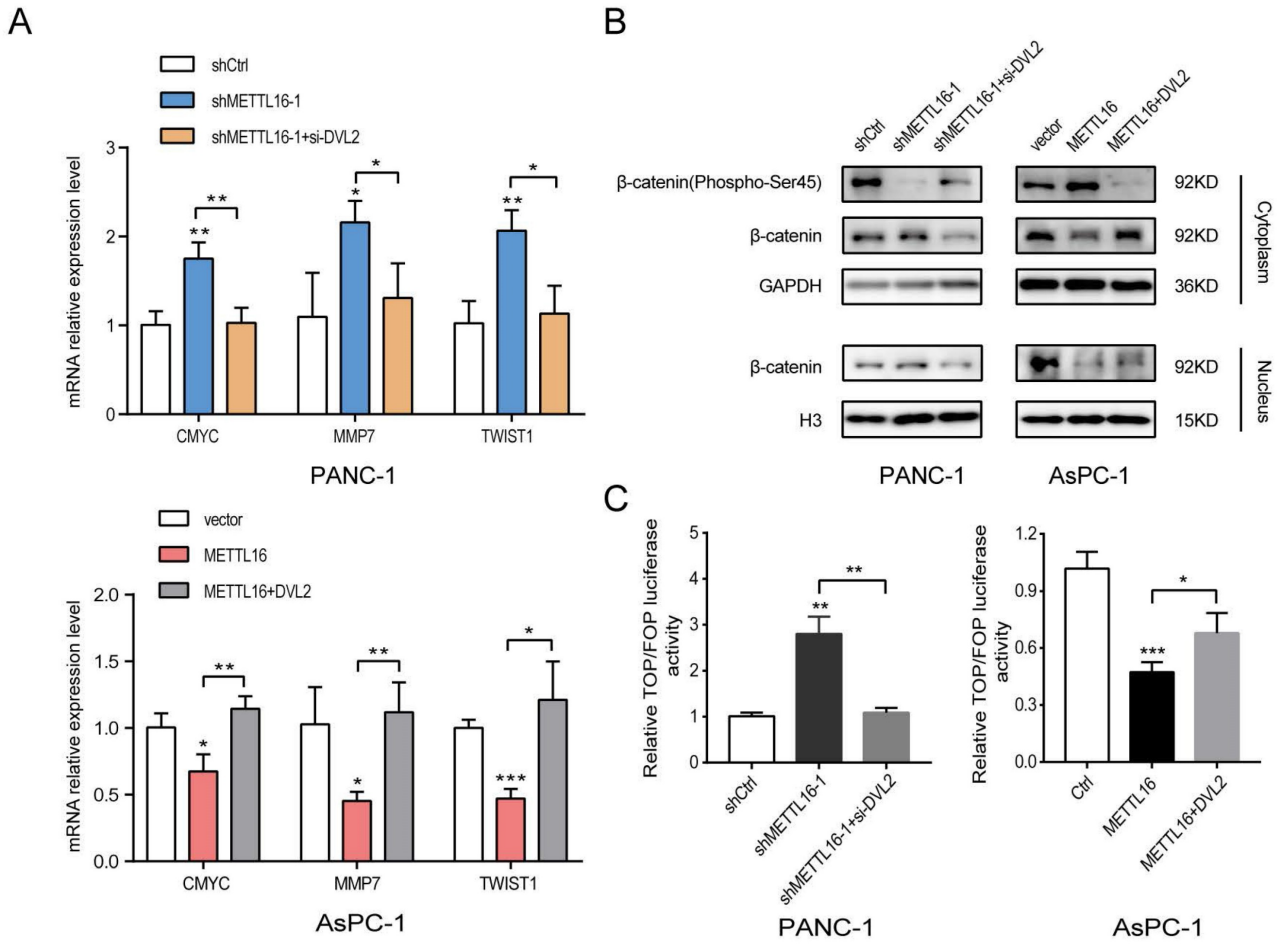
DVL2 is indispensable for the regulation of Wnt/β-catenin signaling by METTL16. A RT-qPCR analysis of the expression of the established downstream targets for the Wnt/β-catenin pathway in the indicated cells. B The expression of β-catenin (Phospho-Ser45), cytosolic β-catenin and nuclear β-catenin in the indicated cells, as determined by western blotting. C The indicated cells were transfected with TOP or FOP reporter and Renilla pRL-TK plasmids and subjected to dual-luciferase assays after transfection. The detected reporter activity was normalized to the Renilla activity. All experiments were performed in triplicate. Data are presented as the mean ± SD (*One-way ANOVA*). *P<0.05, and **P<0.01

To explore whether DVL2 was a contributor to the regulation of Wnt/β-catenin signaling by METTL16, rescue experiments were performed. The expression of DVL2 in PDAC cells after transfection with si-DVL2 or DVL2 overexpression plasmid was measured by western blot (Figure S4C). The results of the western blotting indicated that si-DVL2-2 was more efficient than si-DVL2-1; thus, si-DVL2-2 was chosen for use in the following experiments. The results indicated that DVL2 inhibited the effect of METTL16 on downstream target gene levels in this pathway (Figure 6A). Besides, METTL16-induced alternation of Wnt/β-catenin signaling components was rescued by DVL2 (Figure 6B). Moreover, as illustrated in Figure 6C, DVL2 suppressed the impact of METTL16 on TOP/FOP luciferase activities in PDAC cells (Figure 6C). These results, therefore, confirmed that in PDAC cells, DVL2 acts as a target of METTL16 and mediates its effects.

## Discussion

Approximately 90% of patients are diagnosed at a late stage when they are diagnosed with PDAC, and by 2030 it will be the second leading cause of cancer deaths in the United States, this underscores the need to discover early diagnostic tools and effective therapies^30^, ^31^.

As a reversible process, N6-methyladenosine (m^6^A) modification is prevalent in eukaryotes and is closely associated with a wide range of diseases. In mammalian cells, m^6^A requires writers consisting of METTL3, METTL14, and METTL16, whereas it is reversed by erasers ALKBH5 and FTO, and interacts with readers such as YTHDF1 and IGF2BP1. m^6^A methylation modulates several aspects of mRNA metabolism, pre-mRNA processing, nuclear export, decay, and translation included, and is relevant to the development of numerous diseases, such as cancer^32^, ^33^. For example, METTL3 activated the mA-GLUT1-mTORC1 axis to facilitate colorectal cancer. METTL14 decreases PERP levels to accelerate the development of pancreatic cancer^34^, ^35^. However, the functions of METTL16 in PDAC are just beginning to be understood.

METTL16 is an m^6^A writer consisting of an N-terminal RNA-binding domain, a methyltransferase domain, as well as, vertebrate conserved region (VCR) domains. The N-terminal RNA-binding domain is sufficient to accommodate double-stranded RNA by forming a groove of amino acids. Moreover, the conserved Rossmann-like fold of class I methyltransferases in the methyltransferase domain including the amino acids is almost identical to that in METTL3. However, without the N-terminal domain, it is not enough for that region to bind to RNA^36^, ^37^. Additionally, the domain has a unique “auto-regulatory K-loop” that can weaken the methylation function, which may be related to its function as the sensor of methyl^38^. The VCR region is assumed to interact near the target adenosine on the double-stranded region, allowing better interaction between the adenosine and the methyltransferase domain. Because the VCR region is highly homologous to the KAI domain of TUT1, it is speculated that METTL16 may have a role beyond what is currently known^27^.

METTL16 has recently been confirmed to promote the development of gastric cancer by cyclin D1^39^. In endocrine system tumors, the reduced expression of METTL16 leads to worse survival^40^. Regarding pancreatic cancer, studies have confirmed that METTL16 was downregulated and played a prognostic role for pancreatic cancer patients^41^. Nevertheless, the mechanism by which METTL16 affects the survival of PDAC patients is ambiguous. Rapid disease development and early metastasis cause late diagnosis at the unresectable stage of PDAC patients, additionally, high chemoresistance obstructs effective therapy. As EMT not only is the key step toward metastasis but also acts as the dispensable inducer of chemoresistance^42^, ^43^, we explored the effect of METTL16 on EMT in PDAC. In the present study, the downregulation of METTL16 in PDAC was further verified by IHC analysis of PDAC tissues. In addition, to the best of our knowledge, we demonstrate for the first time that loss of METTL16 could accelerate the EMT of PDAC by activating Wnt/β-catenin signaling.

Initially, METTL16 was assumed to be a ribosomal RNA methyltransferase, but in 2016, Jessica A. Brown, et al. found that METTL16 binds to lncRNA MALAT1^15^. Subsequently, studies indicated that in human embryonic kidney cells, METTL16 modulates alternative splicing of MAT2A RNA in response to the level of SAM. When the SAM level is insufficient, METTL16 only binds to MAT2A without methylation. Inversely, once SAM levels are sufficient, several hairpin loops located in MAT2A 3′UTR are methylated by METTL16, disrupting terminal intron splicing and leading to mRNA degradation. 17. As a catalytic subunit of the splicing mechanism, snRNA U6 was identified to be methylated by METTL16. The VCR domain of METTL16 helps to bend U6 in this process so the adenosine is more accessible to the methyltransferase domain. Some scholars hypothesize that the methylation of snRNA U6 by METTL16 may be related to U6-catalyzed RNA splicing^16^, ^27^. Recently, it has been found that METTL16 is present mainly in the cytoplasm and exerts an m^6^A-independent function by directly binds to the eukaryotic initiation factors 3a and -b, ribosomal RNA through its methyltransferase domain to alter translation and promote the progress of HCC^18^. However, in PDAC cell lines, we illuminated that METTL16 locates in both the nucleus and cytoplasm, but mainly in the nucleus, Thus, we hypothesized that METTL16 mainly plays the role of a methylase in PDAC. Our data indicated that DVL2 is a METTL16 target gene in PDAC in an m^6^A-dependent manner.

DVL2 is a signal-transducing protein involved in Wnt/β-catenin signaling. Research manifested that DVL2 takes part in the regulation of chemoresistance of gliomas via Wnt/β-catenin signaling^44^. In pancreatic cancer, the DVL2/β-catenin pathway was confirmed to facilitate progression mediated by KLF12^29^. In our study, we found that DVL2 is regulated by the m^6^A modification of METTL16. Mechanistically, METTL16 could inhibit the translation of DVL2 mRNA in PDAC. Similarly, the link between enhanced m^6^A and reduced translation capacity has been reported in Slobodin et al.' study^45^.

A recent publication using synthetic activity SAM in METTL16 depletion cells found approximately seventy percent overlap with anti-METTL16-based datasets for METTL16 interactors^46^. It appears that the overlapping substrates are methylated by METTL3. Simply put, METTL16-dependent but not METTL3-dependent m^6^A-containing targets are more likely directly methylated by METTL16. In our study, it was demonstrated that METTL3 or METTL14 cannot reverse the alteration of DVL2 regulated by METTL16; thus, the possibility of METTL16 acting through the METTL3/METTL14 complex is excluded. METTL16 has two verified targets that share a consensus sequence “UACAGARAA”^17^. In addition, in HCC, "UGAAGA" was found to be the top binding motif of METTL16-specific binding transcripts^18^. Therefore, we predict that the methylation site sequence of METTL16 on DVL2 mRNA may be “UACAGARAA” or “UGAAGA”. The latter was found in the DVL2 mRNA sequence, so we performed MeRIP on this sequence (two sites in total). The results indicated that METTL16 binds and methylates the DVL2 mRNA.

In summary, our research demonstrates that downregulation of METTL16 was correlated closely with worse prognosis in patients with PDAC, besides, METTL16-mediated m^6^A modification of DVL2 inhibits metastasis and invasion of PDAC cells by regulating Wnt/β-catenin signaling therefore may provide a promising prognostic factor for PDAC.

## Supplementary Material

Supplementary figures and table.
