# Supplementary material for: Suppression of Wnt/β-catenin Signaling in PDAC via METTL16-mediated N6-methyladenosine Modification of DVL2
Source: J Cancer. 2023 Sep 11;14(16):2964–77. doi: 10.7150/jca.85860 (PMC10583588; doi:10.7150/jca.85860)
Supplement: Supplementary file 1 — Supplementary figures and table. [file jcav14p2964s1.pdf]

Suppression of Wnt/β-catenin signaling in PDAC via METTL16-mediated N6-methyladenosine Modification of DVL2

Lanting Yu[1,2*], Jiawei Lu[1,2*], Ni Xie[1,2*], Lutong Fang[3], Sumin Chen[4], Ying Wu[1,2], Xingpeng Wang[1,2], Baiwen Li[1,2]

1 Department of Gastroenterology, Shanghai General Hospital, Shanghai Jiao Tong University School of Medicine, Shanghai 201620, China

2 Shanghai Key Laboratory of Pancreatic Diseases, Shanghai General Hospital, Shanghai Jiao Tong University School of Medicine, Shanghai 201620, China

3 The First Affiliated Hospital of Anhui Medical University, Anhui 230022, China

4 Department of Gastroenterology, Jiading Branch of Shanghai General Hospital, Shanghai Jiao Tong University School of Medicine, Shanghai 201803, China

[*] Contributed equally

Correspondence to

Professor Baiwen Li, Department of Gastroenterology, Shanghai General Hospital, Shanghai Jiao Tong University School of Medicine, Shanghai, China. Shanghai Key Laboratory of Pancreatic Diseases, Shanghai General Hospital, Shanghai Jiao Tong University School of Medicine, No.650 Fangsong Street, Xinsongjiang Road, Shanghai 201620, China

E-mail: 456@sjtu.edu.en

Professor Xingpeng Wang, Department of Gastroenterology, Shanghai General Hospital, Shanghai Jiao Tong University School of Medicine, Shanghai, China. Shanghai Key Laboratory of Pancreatic Diseases, Shanghai General Hospital, Shanghai Jiao Tong University School of Medicine, No.650 Fangsong Street, Xinsongjiang Road, Shanghai 201620, China

E-mail: richardwangxp@163.com

Supplementary Table S1. Oligos and primers used in this study.

Oligonucleotides	Supplier	Catalog number or sequence
shMETTL16-1	EnzyArtisan	5’-CCCTTGAGACTCAACTATATT-3’
shMETTL16-2	EnzyArtisan	5’-CTCCTGATGGATGCTCTTAAA-3’
si-METTL3	EnzyArtisan	5’-CGUCAGUAUCUUGGGCAAGUUTT-3’
si-METTL14	EnzyArtisan	5’-GCUUACAAAUAGCAACUACAATT-3’
si-DVL2-1	RiboBio	5’-CATGAGCTTTCATCTTACA-3’
si-DVL2-2	RiboBio	5’-TCCACAATGTCTCTCAATA-3’
siR NC #1	RiboBio	Cat# siN0000001-1
DVL2 in pcDNA3.1-C-3*Flag	RiboBio	Cat# ST2204203
PcDNA3.1	RiboBio	Cat# ZT0001
GAPDH forward primer for qPCR	EnzyArtisan	5’-GTCTCCTCTGACTTCAACAGCG -3’
GAPDH reverse primer for qPCR	EnzyArtisan	5’-ACCACCCTGTTGCTGTAGCCAA-3’
METTL16 forward primer for qPCR	EnzyArtisan	5’-TGGAGCAACCTTGAATGGCTGG-3’
METTL16 reverse primer for qPCR	EnzyArtisan	5’-CCATCAGGAGTGTCTTCTGTGG-3’
DVL2 forward primer for qPCR	EnzyArtisan	5’-TCCATACGGACATGGCATCGGT-3’
DVL2 reverse primer for qPCR	EnzyArtisan	5’-CGTGATGGTAGAGCCAGTCAAC-3’
CMYC forward primer for qPCR	EnzyArtisan	5’-GTGCTCCATGAGGAGACACCG-3’
CMYC reverse primer for qPCR	EnzyArtisan	5’-CAGACTCTGACCTTTTGCCAGG-3’
MMP7 forward primer for qPCR	EnzyArtisan	5’-TCGGAGGAGATGCTCACTTCGA-3’
MMP7 reverse primer for qPCR	EnzyArtisan	5’-GGATCAGAGGAATGTCCCATACC-3’
TWIST1 forward primer for qPCR	EnzyArtisan	5’-TGAGCAAGATTCAGACCCTCAAG-3’
TWIST1 reverse primer for	EnzyArtisa	5’-CTGCAGCTTGCCATCTTGGA-3’

qPCR	n	
DVL2 site#1 forward primer for MeRIP- qPCR	EnzyArtisan	5’-GGGGAGACGAAGGTGATTTACC-3’
DVL2 site#1 reverse primer for MeRIP- qPCR	EnzyArtisan	5’-CTGCAGGACGCTCTTGAAATC-3’
DVL2 site#2 forward primer for MeRIP- qPCR	EnzyArtisan	5’-GAGTTTCTTACATTTTTGGGACTTT-3’
DVL2 site#2 reverse primer for MeRIP- qPCR	EnzyArtisan	5’-GGCTGTGGGTAACTGGAGG-3’

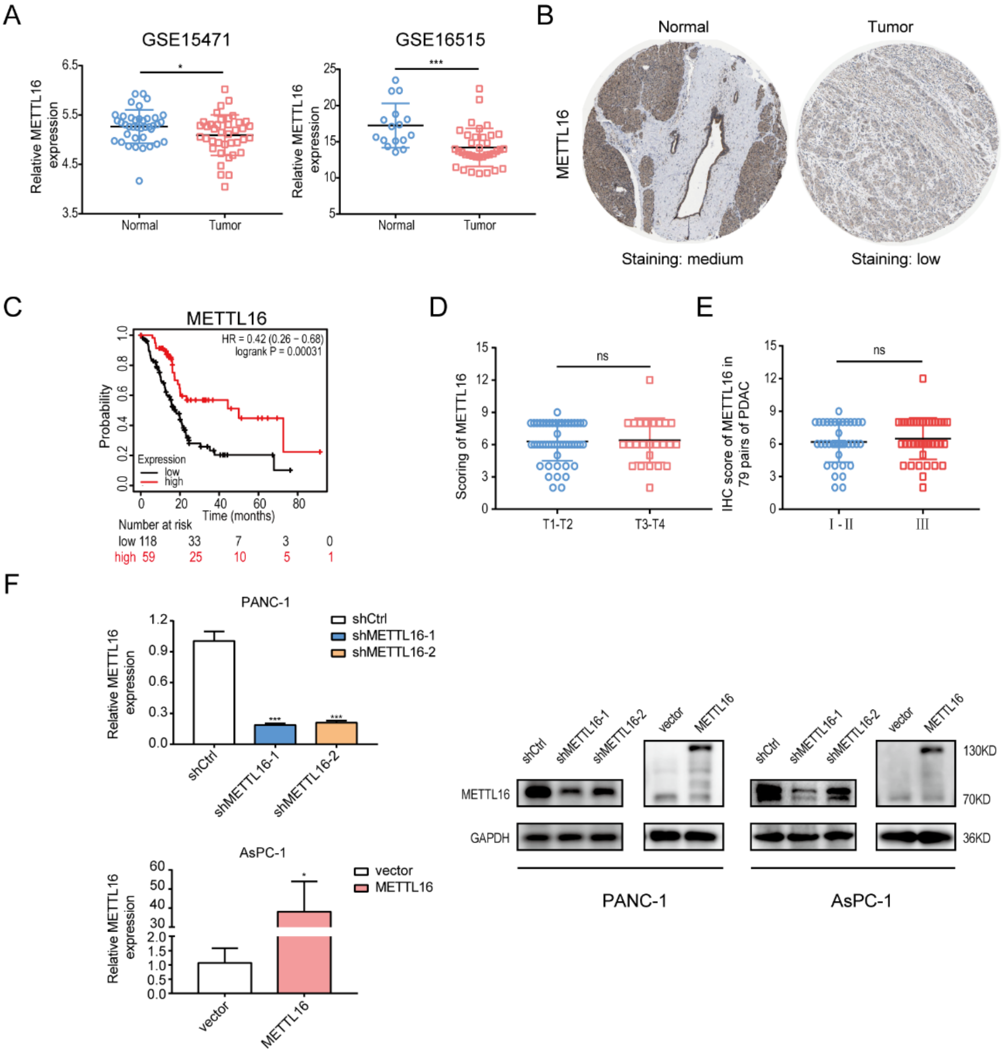

Supplementary Figure 1

A METTL16 mRNA expression levels in Gene Expression Omnibus datasets, GSE15471 (N, n=39; T, n=39) and GSE16515 (N, n=16; T, n=36). B The protein expression of METTL16 in pancreatic cancer tissue through analysis of HPA databases. C Kaplan-Meier curves for the overall survival probability in 178 patients with PDAC with low (n=118) and high (n=59) METTL16 expression levels. Curves were produced using a Kaplan-Meier plotter and analyzed using the log-rank test. D METTL16 expression in TMAs with different T stage. E METTL16 expression in TMAs with different pathological grade. F RT-qPCR and western blotting were used to determine METTL16 expression levels following transduction with shMETTL16 or METTL16 overexpression lentiviruses. All experiments were performed in triplicate. Data are presented as the mean ± SD (*One-way ANOVA* and *Student's t-test*). *P<0.05, **P<0.01, and ***P<0.001.

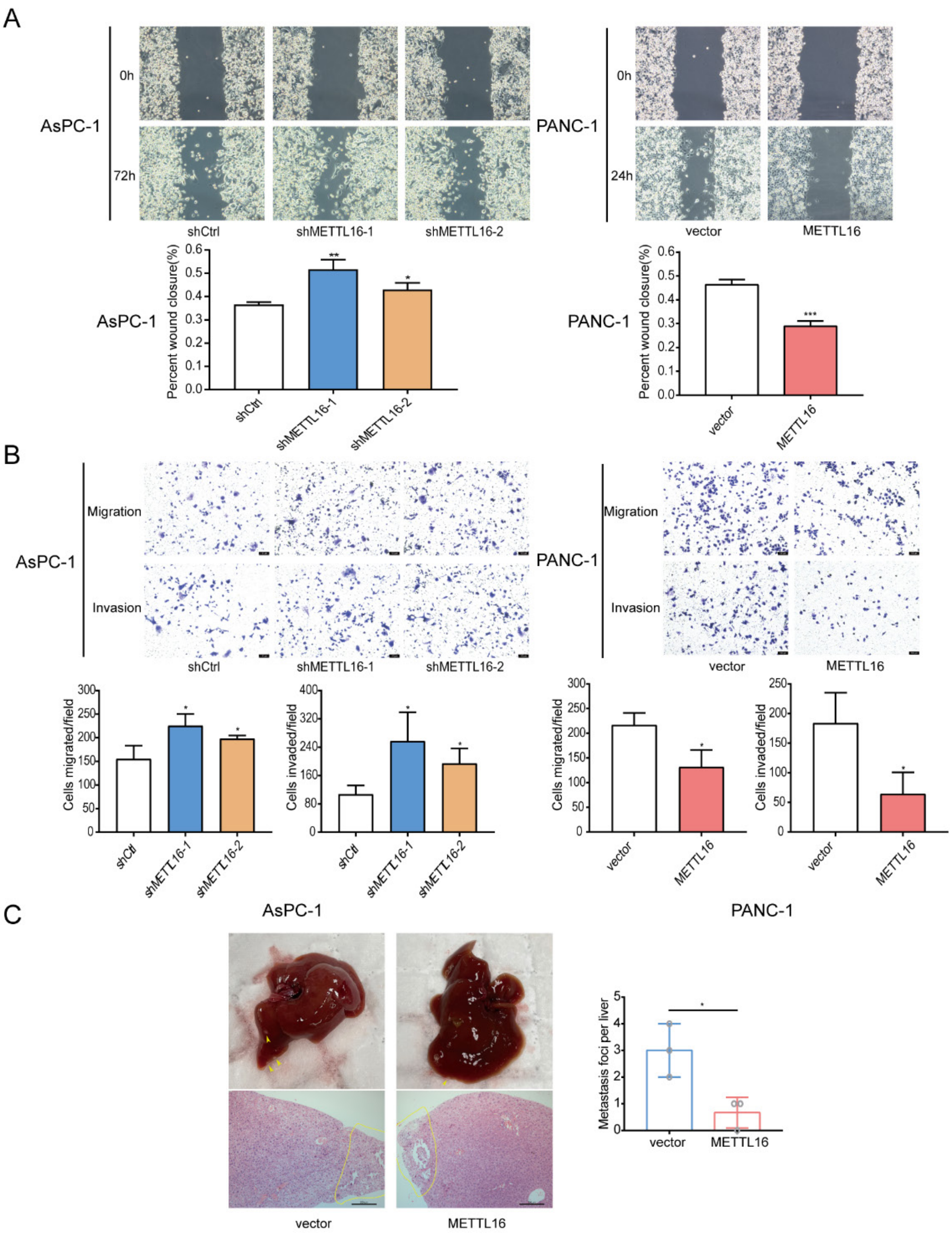

Supplementary Figure 2

A and B Cell migration and invasion abilities were detected in PDAC cells transduced with shMETTL16 or METTL16 lentiviruses by wound-healing assays and transwell assays. C Representative liver images and H&E staining of the liver tissues of the respective groups. All experiments were performed in triplicate. *P<0.05, **P<0.01, and ***P<0.001. All the data are presented as mean ± SD (*One-way ANOVA* and *Student's t-test*).

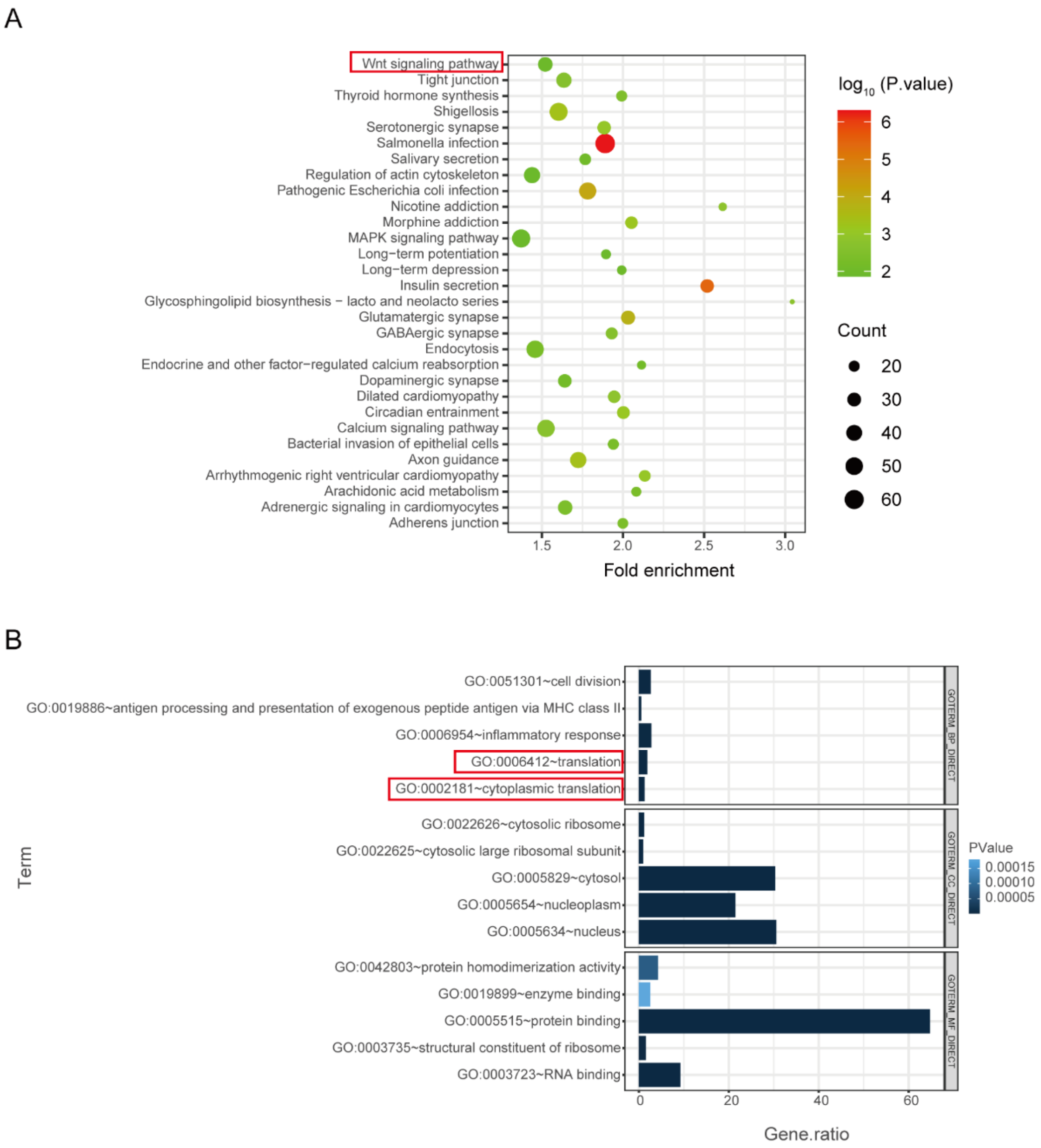

Supplementary Figure 3

A The KEGG pathway analysis was conducted on METTL16 co-expressed genes in TCGA PAAD by the DAVID database. B GO analysis comprising cellular component (CC), biological process (BP) and molecular function (MF) was conducted on METTL16 co-expressed genes in TCGA PDAC

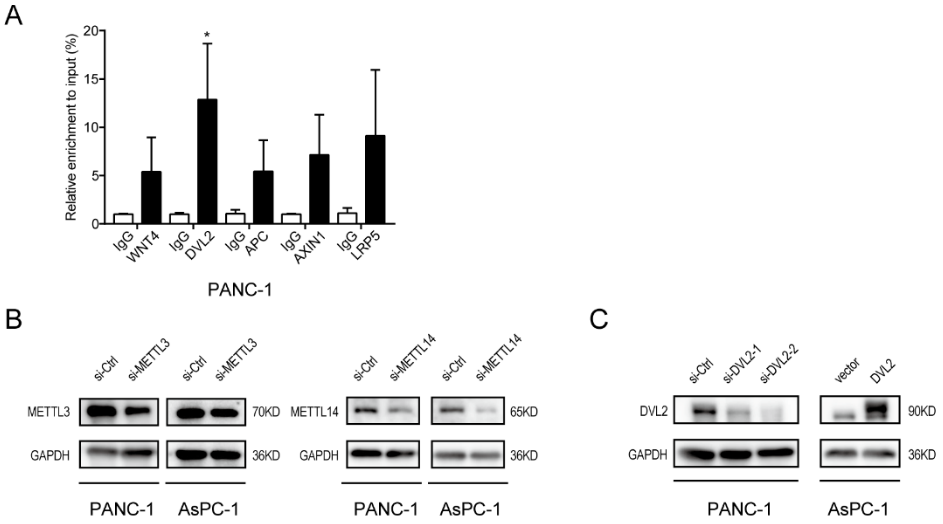

Supplementary Figure 4

A RIP-qPCR analysis of binding of METTL16 protein to mRNA of WNT4, DVL2, APC, AXIN1 and LRP5. B The expression levels of METTL3 or METTL14 were evaluated through western blotting after PDAC cells were transfected with si-METTL3 or si-METTL14. C The expression levels of DVL2 were evaluated through western blotting after PDAC cells were transfected with si-DVL2. All experiments were performed in triplicate. Data are presented as the mean ± SD (*Student's t-test*). *P<0.05
